# The Effects of Sodium-Glucose Cotransporter 2 Inhibitors on Sympathetic Nervous Activity

**DOI:** 10.3389/fendo.2018.00421

**Published:** 2018-07-26

**Authors:** Ningning Wan, Asadur Rahman, Hirofumi Hitomi, Akira Nishiyama

**Affiliations:** Department of Pharmacology, Faculty of Medicine, Kagawa University, Kagawa, Japan

**Keywords:** sodium-glucose cotransporter 2 (SGLT2) inhibitor, EMPA-REG OUTCOME trial, CANVAS program, blood pressure, heart rate, sympathetic nervous activity

## Abstract

The EMPA-REG OUTCOME study revealed that a sodium-glucose cotransporter 2 (SGLT2) inhibitor, empagliflozin, can remarkably reduce cardiovascular (CV) mortality and heart failure in patients with high-risk type 2 diabetes. Recently, the CANVAS program also showed that canagliflozin, another SGLT2 inhibitor, induces a lower risk of CV events. However, the precise mechanism by which an SGLT2 inhibitor elicits CV protective effects is still unclear. Possible sympathoinhibitory effects of SGLT2 inhibitor have been suggested, as significant blood pressure (BP) reduction, following treatment with an SGLT2 inhibitor, did not induce compensatory changes in heart rate (HR). We have begun to characterize the effects of SGLT2 inhibitor on BP and sympathetic nervous activity (SNA) in salt-treated obese and metabolic syndrome rats, who develop hypertension with an abnormal circadian rhythm of BP, a non-dipper type of hypertension, and do not exhibit a circadian rhythm of SNA. Treatment with SGLT2 inhibitors significantly decreased BP and normalized circadian rhythms of both BP and SNA, but did not change HR; this treatment was also associated with an increase in urinary sodium excretion. Taken together, these data suggest that an SGLT2 inhibitor decreases BP by normalizing the circadian rhythms of BP and SNA, which may be the source of its beneficial effects on CV outcome in high-risk patients with type 2 diabetes. In this review, we briefly summarize the effects of SGLT2 inhibitors on BP and HR, with a special emphasis on SNA.

## Introduction

Sodium-glucose cotransporter 2 (SGLT2) is located at the S1 and S2 segments of the proximal tubule epithelium, which reabsorbs approximately 90% of filtered glucose (1). SGLT2 inhibitors induce glycosuria (2) and are widely used as antihyperglycemic agents in patients with type 2 diabetes (3). Recently, the EMPA-REG OUTCOME study demonstrated that treatment with empagliflozin, an SGLT2 inhibitor, significantly decreased the primary composite outcome of cardiovascular (CV) events, thereby reducing CV mortality by 38% (4). Further studies have shown that empagliflozin reduced heart failure hospitalization and CV death, with a consistent benefit in patients with and without baseline heart failure (5). The CANVAS program has also shown that canagliflozin, another SGLT2 inhibitor, lowers the risk of CV events by providing renal protection in type 2 diabetes patients (6). Moreover, in the large, multinational CVD-REAL study, treatment with an SGLT2 inhibitor was associated with lower rates of hospitalization for heart failure and death, compared with other glucose lowering drugs, implying CV benefits from SGLT2 inhibitor usage (7). The underlying mechanism by which an SGLT2 inhibitor improves CV disease is not clear; however, which the mechanism may not be limited to effects on metabolic parameters, body weight, and blood pressure (BP) (4).

There were close links and interactions between sympathetic nervous activity (SNA) and metabolic syndrome (8). And patients with obesity, hypertension, or diabetes exhibit high CV risk, which is associated with an inappropriate augmentation of SNA (9). A systematic meta-analysis revealed that SGLT2 inhibitors decrease systolic blood pressure (SBP) and diastolic blood pressure (DBP) from baseline (−4.0 mmHg, and −1.6 mmHg, respectively) (2). However, clinical trials have failed to show notable changes or compensatory increases in heart rate (HR), following the administration of SGLT2 inhibitors (2, 10). These data suggest a possible sympathoinhibitory effect from an SGLT2 inhibitor, which may contribute in part to the cardioprotective effects of SGLT2 inhibitor therapy. In this review, we briefly summarize the effects of SGLT2 inhibitors on BP and HR in patients with type 2 diabetes. We also discuss the hypothesis that SGLT2 inhibitors elicit SNA inhibition.

## Effects of SGLT2 inhibitors on BP

Most clinical studies have shown that treatment with SGLT2 inhibitors, either as mono- or add-on therapies, significantly decreases both SBP and DBP in patients with type 2 diabetes (Table 1); however, some studies have shown no notable change in DBP (3, 23). Meta-analyses have revealed that SGLT2 inhibitors induce statistically significant reductions in SBP and DBP (2, 39). And Reed et al. (10) showed reasonable explanation of BP-lowering effects of SGLT2 inhibitors in type 2 diabetes. Interestingly, the extent of antihypertensive efficacy for each SGLT2 inhibitor differs according to patient background. For example, in a study of 1,031 type 2 diabetic patients who were divided into 5 groups based on body mass index (BMI, kg/m^3^) level [low-to-medium (<22.5, *n* = 222); medium (22.5–24.9, *n* = 270); high-level 1 (25–27.4, *n* = 262); high-level 2 (27.5–29.9, *n* = 142); and very-high (≥30, *n* = 135)], treatment with luseogliflozin significantly decreased SBP and DBP, relative to baseline, in all groups. However, reductions in SBP and DBP were greater in groups with higher BMI levels (40), suggesting that an SGLT2 inhibitor effectively decreases BP in high BMI, type 2 diabetic patients. Another clinical trial with ipragliflozin (50 mg/day for 24 weeks) showed no significant change in BP in 50 patients with type 2 diabetes. However, in 23 patients with poorly controlled BP (SBP ≥ 140 mmHg and/or DBP ≥ 90 mmHg), treatment with ipragliflozin significantly reduced SBP and DBP (−6.6 mmHg and, −3.0 mmHg, respectively) (41). Similarly, treatment with empagliflozin for 12 weeks resulted in a greater BP reduction in hypertensive patients with type 2 diabetic who exhibited higher baseline BP (17). Taken together, these results suggest that SGLT2 inhibitors are effective for BP reduction in poorly controlled hypertensive patients with type 2 diabetes.

**Table 1:** Effects of SGLT2 inhibitors on blood pressure and heart rate.

**Authors**	**Study design**	**Treatment period**	**Drugs and doses**	**SBP (mmHg)**		**DBP (mmHg)**		**HR (bpm)**	
				**Baseline**	**Change**	**Baseline**	**Change**	**Baseline**	**Change**
Cherney et al. 11	Clinical studies T1D	8 weeks	Empagliflozin 25 mg	112.1 (8.9)	−1.5	65.2 (8.3)	−1.4	72.0 (11.0)	−1.2
Häring et al. 12	Clinical studies T2D	24 weeks	Empagliflozin 10 mg 25 mg	128.7 129.3	−4.1 −3.5	78.4 79	−2.1 −2.2	NR	No change
Chilton et al. 13	Clinical studies T2D	12 weeks 24 weeks	Empagliflozin 10/25 mg	NR	−3.9 −1.5	NR	−3.6 −1.3	NR	−0.6 −0.8
Kovacs et al. 14	Clinical studies T2D	24 weeks	Empagliflozin 10 mg 25 mg	126.5 126.0	−3.14 −4.00	77.2 77.2	−1.49 −2.21	NR	No change
Nishimura et al. 15	Clinical studies T2D	4 weeks	Empagliflozin 10 mg 25 mg	119.1 (15.9) 124.0 (11.6)	−4.9 −5.9	70.7 (10.7) 74.7 (8.0)	−1.3 −5.4	65.3 (8.7) 64.6 (7.8)	0.2 (4.8) −1.7 (5.8)
Tikkanen et al. 16	Clinical studies T2D	24 weeks	Empagliflozin 10 mg 25 mg	129.6 130.0	−4.5 (0.7) −5.2 (0.7)	79.6 78.4	−2.0 (0.5) −1.6 (0.5)	NR	No change
Rosenstock et al. 17	Clinical studies T2D with hypertension	12 weeks	Empagliflozin 10 mg 25 mg	131.34 131.18	−2.95 −3.68	75.13 74.64	−1.04 −1.40	NR	−0.17 (7.70) −0.74 (6.16)
Rosenstock et al. 18	Clinical studies T2D	78 weeks	Empagliflozin 10 mg 25 mg	132.4 132.8	−4.1 −2.4	78.4 77.9	−2.9 −1.5	NR	No change
Rosenstock et al. 19	Clinical studies T2D	52 weeks	Empagliflozin 10 mg 25 mg	134.2 (16.4) 132.9 (14.2)	−3.4 −3.8	79.5 (8.5) 78.7 (8.5)	−1.2 −2.5	NR	No change
			Mono						
Ferrannini et al. 20	Clinical studies T2D	78 weeks	Empagliflozin 10 mg	131.6	0.1	79.5	−1.6	NR	No change
			25 mg	131.9	−1.7	80.2	−2.2		
			Add-on						
			Empagliflozin 10 mg	133.9	−3.3	80.7	−0.9		
			25 mg	134.5	−3.0	81.2	−2.0		
Wilding et al. 21	Clinical studies T2D	104 weeks	Dapagliflozin 5–10 mg 10 mg	NR	−2.6 −2.9	NR	−7.5 −4.0	NR	−1.3 −1.2
Nauck et al. 22	Clinical studies T2D	52 weeks	Add-on						
			Dapagliflozin 2.5–10 mg	132.8	−4.3	80.6	−1.6	74.1 (10.9)	−0.1 (0.5)
List et al.^23^	Clinical studies T2D	12 weeks	Dapagliflozin 2.5 mg 5 mg 10 mg 20 mg 50 mg	127 (14) 126 (13) 127 (16) 127 (15) 126 (16)	−3.1 (10.7) −2.9 (12.7) −6.4 (11.4) −4.3 (12.3) −2.6 (13.1)	78 (8) 76 (8) 77 (8) 77 (8) 77 (9)	0.8 (6.4) −0.3 (7.0) −2.6 (7.7) −0.5 (7.1) 0.1 (8.0)	71 (10) 70 (10) 69 (8) 68 (10) 70 (10)	−1.4 (8.0) −1.0 (8.9) −0.03 (8.9) 1.9 (11.2) −2.3 (7.1)
Sjöström et al. 24	Clinical studies T2D in hypertensive T2D in non–hypertensive	24 weeks	Dapagliflozin 10 mg	149.9 (7.8) 124.3 (10.8)	−3.6 −2.6	83.5 (9.1) 76.4 (8.3)	−1.2 −1.2	NR	−0.5 0.1
Wilding et al. 25	Clinical studies T2D	48 weeks	Dapagliflozin 2.5 mg 5 mg 10 mg	139.6 (17.7) 137.8 (16.2) 140.6 (16.7)	−5.30 −4.33 −4.09	79.5 (10.1) 81.1 (8.9) 79.9 (9.3)	−2.96 −2.64 −2.85	75.4 (11.9) 73.9 (11.1) 74.8 (11.2)	−1.44 −1.25 −0.84
Cefalu et al. 26	Clinical studies T2D	52 weeks	Canagliflozin 100 mg 300 mg	130.0 (12.4) 130.0 (13.8)	−3.3 −4.6	78.7 (8.0) 79.2 (8.4)	−1.8 −2.5	74.2 74.6	−1.1 (8.5) −1.2 (8.7)
Devineni et al. 27	Clinical studies T2D	4 weeks	Canagliflozin 100 mg 300 mg	NR	−10.7 (9.0) −8.8 (12.4)	NR	−7.1 (4.5) −3.3 (6.1)	NR	No change
Rosenstock et al. 28	Clinical studies T2D	12 weeks	Canagliflozin 50 mg 100 mg 200 mg 300 mg BID 300 mg	126.8 126.5 124.3 126.1 128.5	−1.3 1.0 −2.1 −4.9 −3.6	76.9 77.6 77.4 79.8 78.9	−0.1 −0.2 −1.7 −2.1 −2.4	69.9 71.0 70.8 72.6 71.8	−0.2 −0.2 0.6 −1.7 0.2
Leiter et al. 29	Clinical studies T2D	104 weeks	Canagliflozin 100 mg 300 mg	130.0 (12.4) 130.0 (13.8)	−2.0 −3.1	78.7 (8.0) 79.2 (8.4)	−1.3 −2.2	NR	−0.1 −0.2
Sha et al. 30	Clinical studies T2D	2 weeks	Canagliflozin 30 mg 100 mg 200 mg 400 mg BID 300 mg	125.6 (17.7) 130.8 (10.4) 120.3 (5.1) 122.2 (14.7) 125.3 (14.9)	−10.9 (15.5) −4.7 (7.3) −11.5 (7.3) −9.4 (7.2) −9.8 (7.6)	74.6 (7.8) 78.7 (7.1) 72.7 (5.3) 71.7 (6.5) 74.4 (6.8)	−3.9 (6.8) 0.2 (6.6) −4.5 (6.1) −3.4 (5.1) −2.9 (4.5)	71.5 (13.7) 73.2 (7.9) 68.0 (7.4) 71.2 (6.0) 68.1 (7.0)	−7.1 (10.4) −9.7 (5.6) −5.1 (6.1) −4.9 (6.0) −5.5 (3.9)
L-González et al. 31	Clinical studies T2D	52 weeks	Canagliflozin 100 mg 300 mg	128.0 (12.7) 128.7 (13.0)	−3.5 −4.7	77.7 (8.4) 77.9 (8.3)	−1.8 −1.8	NR	−1.3 −1.9
Stenlöf et al. 32	Clinical studies T2D	26 weeks	Canagliflozin 100 mg 300 mg	126.7 (12.5) 128.5 (12.7)	−3.3 −5.0	77.7 (6.8) 79.1 (8.3)	−1.7 −2.1	NR	−1.6 −0.5
Wilding et al. 33	Clinical studies T2D	52 weeks	Canagliflozin 100 mg 300 mg	130.4 (13.5) 130.8 (12.8)	−3.1 −2.9	78.2 (8.3) 78.9 (8.1)	−2.2 −1.7	NR	−1.2 −0.4
Schernthaner et al. 34	Clinical studies T2D	52 weeks	Canagliflozin 300 mg	137.2 (13.2)	−5.1	79.2 (7.8)	−3.0	NR	−0.1
Forst et al. 35	Clinical studies T2D	26 weeks 52 weeks	Canagliflozin 100 mg 300 mg Canagliflozin 100 mg 300 mg	126.4 (12.3) 126.7 (12.0) 126.4 (12.3) 126.7 (12.0)	−5.3 −4.7 −3.4 −3.7	75.6 (7.8) 76.6 (8.5) 75.6 (7.8) 76.6 (8.5)	−3.3 −3.5 −2.5 −2.7	NR NR	−0.3 −1.3 0.5 −1.0
Yale et al. 36	Clinical studies T2D with CKD	26 weeks	Canagliflozin 100 mg 300 mg	135.9 (13.1) 136.7 (15.0)	−6.1 −6.4	73.5 (8.8) 75.7 (7.8)	−2.6 −3.5	NR	−1.9 −1.1
Rahman et al. 37	Animal studies Metabolic syndrome rats	5 weeks	Luseogliflozin 10 mg/kg	NR	Reduction	NR	NR	NR	No change
Maegawa et al. 38	Clinical studies T2D	3 months	Ipragliflozin 25–100 mg	133.4 (15.2)	−4.1	78.2 (11.0)	−2.2	77.3 (12.0)	−0.9

*Data are expressed as mean (standard deviation) or mean. SBP means systolic blood pressure; DBP means diastolic blood pressure; HR means heart rate; bpm means beats per minute; T1D means type 1 diabetes; T2D means type 2 diabetes; CKD means chronic kidney disease; NR means no report*.

## Effects of SGLT2 inhibitors on dipping pattern of BP

The restoration and maintaining a normal circadian rhythm is crucial to CV health (42). Diminished nocturnal decline in BP has been established as an important determinant for CV risk, independent of overall BP during a 24-h period (43). We have recently shown that SGLT2 inhibitors improve disrupted circadian rhythms of BP in metabolic syndrome rats [SHR/NDmcr-cp(+/+) rats; SHRcp] (37) and salt-treated obese Otsuka Long Evans Tokushima Fatty (OLETF) rats (44), both of which show non-dipper type of hypertension. Rahman et al. (37) showed a significant BP-lowering effect from luseogliflozin therapy in SHRcp rats. Interestingly, significant differences in BP levels appeared between dark and light periods, following treatment with an SGLT2 inhibitor, suggesting that the SGLT2 inhibitor altered the circadian rhythm of SBP, from a non-dipper type to a dipper type. Similar effects were reported by Takeshige et al. (44) in salt-treated obese OLETF rats, following use of another SGLT2 inhibitor, empagliflozin. In these obese animals, high salt treatment increased BP and abolished differences in BP between dark and light periods, suggesting a non-dipper type of hypertension. Treatment with empagliflozin prevented the development of salt-induced hypertension and reversed their circadian rhythm of BP, from a non-dipper pattern to a dipper pattern. In SHRcp (37) and salt-treated obese rats (44), SGLT2 inhibitor-induced normalization of disrupted circadian rhythm of BP was associated with increased urinary excretion of sodium. Overall, these data suggest that an SGLT2 inhibitor induces natriuresis, which plays an important role in the improvement of the circadian rhythm of BP in type 2 diabetes (45).

Recently, a clinical case study examined the effect of dapagliflozin (5 mg/day) in patients with type 2 diabetes who exhibited a non-dipper type (sleep-time mean SBP > 90% of awake-time mean) of hypertension. Administration of dapagliflozin significantly decreased BP and altered the circadian dipping pattern of BP, from a non-dipper type to a dipper type (sleep-time mean SBP ≤ 90% of awake-time mean) (46). Another empagliflozin clinical trial also revealed that the reduction in BP was greater during sleep-time, than during wake-time, in type 2 diabetes patients with non-dipper hypertension (47). These data indicate that BP reduction by an SGLT2 inhibitor is associated with restoration of a disrupted circadian rhythm of BP, from a non-dipper pattern to a dipper pattern, in hypertensive patients with type 2 diabetes.

## Effects of SGLT2 inhibitors on HR

As shown in Table 1, many clinical studies have investigated the effects of SGLT2 inhibitors on BP and HR in patients with type 2 diabetes. Many clinical trials have shown that SGLT2 inhibitors significantly decrease BP in patients with type 2 diabetes; however, no study has reported any meaningful change or compensatory increase in HR. We have also recently monitored BP and HR, using a telemetry system, in hypertensive animals. We found that luseogliflozin significantly decreased BP, but did not change HR, in SHRcp rats (37). Recently, Sano et al. (48) reviewed clinical data regarding luseogliflozin treatment in Japanese patients with type 2 diabetes; their report showed that luseogliflozin significantly decreased HR in patients with high baseline HR levels (≥ 70/min before treatment). The authors of that study hypothesize that reduction in HR, by treatment with an SGLT2 inhibitor, is induced by the sympathoinhibitory effect of an SGLT2 inhibitor, in these patients.

## Effects of SGLT2 inhibitors on SNA

As discussed above, both clinical and animal studies indicate that SGLT2 inhibitors decrease BP without changing HR. The absence of HR changes, along with the reduction in BP, supports the notion that SGLT2 inhibitors elicit inhibitory effects on SNA; importantly, SNA strongly correlates with CV mortality (49). Previous studies have revealed that an SGLT2 inhibitor decreases SNA: Chiba et al. (50) showed that acute administration of dapagliflozin significantly suppressed norepinephrine turnover in brown adipose tissue of mice, which reflects SNA in brown adipose tissue. Further, Yoshikawa et al. (51) assessed the effects of ipragliflozin on arterial pressure and low frequency (LF, 0.04–0.60 Hz) of systolic arterial pressure, which reflects the level of sympathetic vasoconstrictor activity, in diabetes mellitus rats; their study demonstrated that inhibition of SGLT2 attenuated the arterial pressure lability associated with sympathoinhibition during the working period. Matthews et al. (52) concluded that SNA was upregulated in obesity and type 2 diabetes, and showed that dapagliflozin reduced SNA markers, such as tyrosine hydroxylase and noradrenaline, in the kidney and heart of C57BL6/J mice; these markers were routinely elevated by high-fat diet treatment. A rising in muscle SNA is usual during hypovolemia, like diuretic effects (53). Jordan et al. (54) demonstrated that there was no significant changes in muscle SNA despite increases in urine volume after short-term treatment of empagliflozin in type 2 diabetes, which suggested a possible inhibitory effects of SGLT2 inhibitor on SNA. However, Kusaka et al. (55) utilized a telemetry system to show that empagliflozin did not elicit significant changes in averaged 24-h SBP, DBP, or HR in SHRcp rats. They also measured LF (0.25–0.75 Hz) of SBP, and showed that treatment with empagliflozin did not alter LF of SBP, or its circadian rhythm, in those animals. Recently, Rahman et al. (37) showed that treatment with luseogliflozin tended to decrease the LF of SBP in SHRcp rats, but these were not statistically significant changes. However, when the LF of SBP was separately analyzed during dark (working) and light (sleeping) time periods, the investigators found that luseogliflozin significantly decreased LF of SBP only during the sleeping period, but not during the working period.

**Figure 1:**
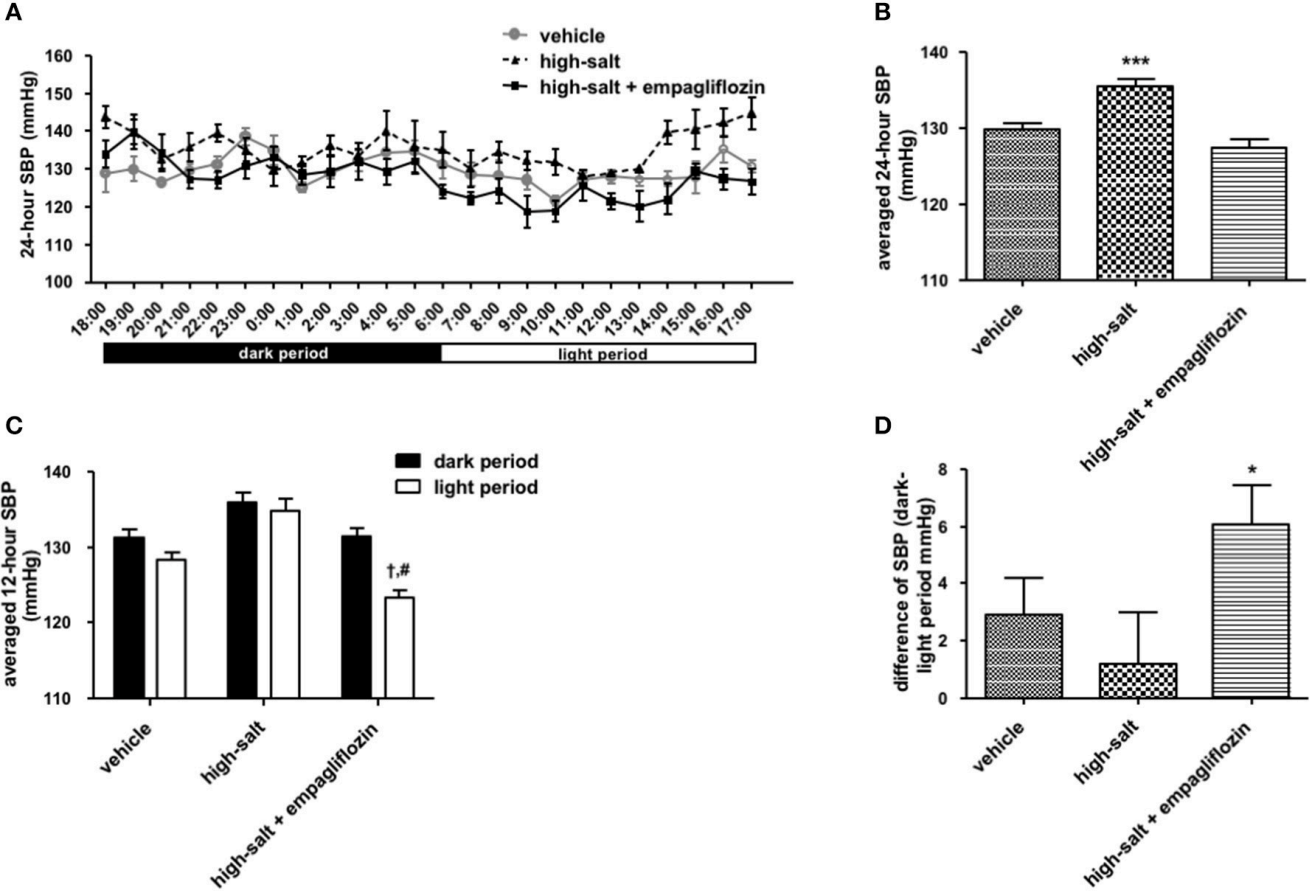
Effects of empagliflozin treatment on systolic blood pressure (SBP), and on circadian rhythm of SBP, in Otsuka Long Evans Tokushima Fatty (OLETF) rats. **(A)** 24-h SBP. **(B)** Average of 24-h SBP. **(C)** SBP in dark and light periods. **(D)** Differences between dark and light period in SBP. OLETF rats were treated with vehicle (vehicle, *n* = 7), 1% NaCl drinking water (high-salt, *n* = 5), or 1% NaCl drinking water and empagliflozin (high-salt + empagliflozin, *n* = 8), for 5 weeks. Values are mean ± SEM. ^***^P < 0.0001 vs. vehicle and high-salt + empagliflozin (one-way analysis of variance followed by Tukey's multiple comparison test), †*P* < 0.0001 vs. high-salt + empagliflozin dark period (2-way analysis), #*P* < 0.0001 vs. high-salt light period (*t*-test), ^*^P < 0.05 vs. high-salt (*t*-test).

**Figure 2:**
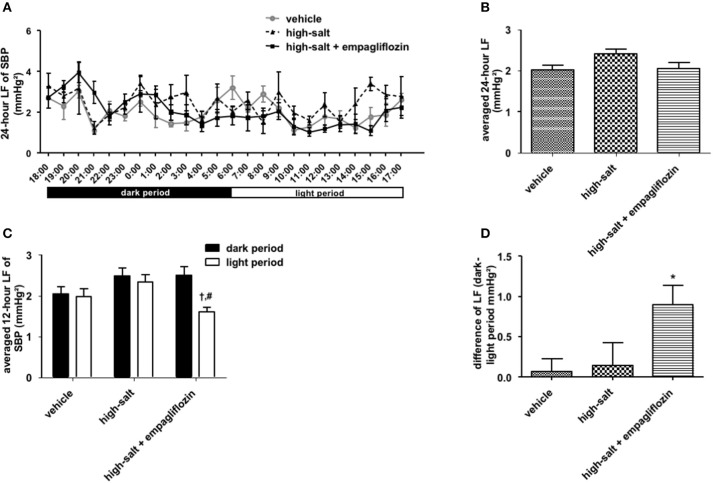
Effects of empagliflozin treatment on low frequency (LF) of systolic blood pressure (SBP), and on circadian rhythm of LF of SBP, in Otsuka Long Evans Tokushima Fatty (OLETF) rats. **(A)** 24-h LF of SBP. **(B)** Average of 24-h LF of SBP. **(C)** LF of SBP in dark and light period. **(D)** Differences between dark and light period in LF of SBP. OLETF rats were treated with vehicle (vehicle, *n* = 7), 1% NaCl drinking water (high-salt, *n* = 5), or 1% NaCl drinking water and empagliflozin (high-salt + empagliflozin, *n* = 8), for 5 weeks. Values are mean ± SEM. †*P* < 0.001 vs. high-salt + empagliflozin dark period (2-way analysis), #*P* < 0.001 vs. high-salt light period (*t*-test), ^*^P < 0.05 vs. vehicle (one-way analysis of variance followed by Tukey's multiple comparison test).

To confirm whether the sympathoinhibitory effect of an SGLT2 inhibitor is dependent on its class-effect or drug-effect, similar experiments were performed to examine the effects of another SGLT2 inhibitor, empagliflozin, in obese OLETF rats. Twenty male OLETF rats (13 weeks old) were implanted with radiotelemetry devices. After 2 weeks of acclimatization, animals were treated with vehicle (0.5% carboxymethylcellulose, *n* = 7), high salt (1% NaCl in drinking water, *n* = 5), or high salt plus empagliflozin (10 mg/kg per day, *n* = 8), for 5 weeks. We analyzed the 24-h SBP (Figure 1) and LF (0.25–0.75 Hz) of SBP (Figure 2), respectively; we found that high salt treatment significantly increased 24-h SBP, while empagliflozin inhibited this salt-induced increase in SBP (Figures 2A, B). Interestingly, differences in BP between dark and light periods were not observed in high salt-treated obese animals, suggesting a lack of circadian rhythm of BP in these animals. However, obvious circadian rhythms of SBP appeared upon administration of empagliflozin to high salt-treated obese rats (Figures 1C,D). Conversely, empagliflozin did not change HR (data not shown). Empagliflozin also tended to decrease the 24-h averaged LF of SBP; however, differences among the groups were not statistically significant (Figures 2A,B). Further, empagliflozin significantly decreased LF of SBP only during the sleeping period, and differences between working and sleeping periods were elevated. Consequently, circadian rhythms in the LF of SBP were quite clear after empagliflozin administration in high salt-treated obese rats (Figures 2C,D). These results support the hypothesis that inhibition of SGLT2 improves the circadian rhythm of SNA through its sympathoinhibitory class-effect during the sleeping period.

## Conclusions

**Figure 3:**
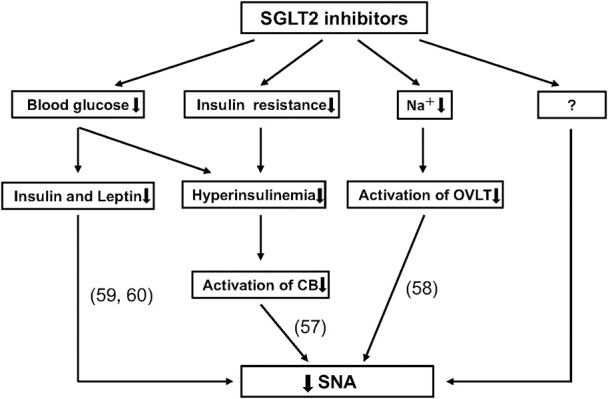
Possible mechanisms for reducing sympathetic nervous activity (SNA) through use of sodium-glucose cotransporter 2 (SGLT2) inhibitors. Recent studies have suggested that SGLT2 inhibitors elicit a reduction in SNA by decreasing insulin, leptin (59, 60) and blood glucose levels; and by improving insulin resistance and hyperinsulinemia, which could reduce the activation of carotid body (CB) (57); as well as by reducing sodium volume, which inhibits the activation of organum vasculosum laminae terminalis (OVLT) (58). Importantly, there are likely to be other mechanisms that have not been described.

Here, we have summarized clinical data regarding the effects of SGLT2 inhibitors on BP and HR in patients with type 2 diabetes. During treatment with an SGLT2 inhibitor, BP reduction is not accompanied by compensatory increases or notable changes in HR. Further, SGLT2 inhibitors exhibit beneficial influences on the circadian rhythms of BP and SNA. Thus, these effects of SGLT2 inhibitors may be important in their CV protective effects, as shown in the EMPA-REG OUTCOME and CANVAS programs (4–6). The precise mechanism by which an SGLT2 inhibitor normalizes disrupted circadian rhythms of BP and SNA is not clear; however, multiple processes may be involved, including reduction of blood glucose level and body weight, improvement of insulin resistance, and initiation of natriuresis (8, 56–60) (Figure 3). Further studies are necessary to determine the mechanism responsible for the effects of SGLT2 inhibitors on SNA.

## Author contributions

NW and AN analyzed previous clinical data. NW and AR performed the animal experiments and analyzed all experimental data. NW, HH, and AN wrote the manuscript. AN and HH supervised the study and revised the manuscript. All authors have read and approved the final manuscript.

### Conflict of interest statement

Empagliflozin was provided by Boehringer Ingelheim Co., Ltd. (to AN). This is a collaborative study, in part, with Boehringer Ingelheim Co., Ltd.
